# Porous Organic Cages for CO_2_ Capture and Confined Reduction

**DOI:** 10.1002/anie.5880155

**Published:** 2026-05-28

**Authors:** Valeria Amendola, Sonia La Cognata

**Affiliations:** ^1^ Department of Chemistry University of Pavia Pavia Italy; ^2^ INSTM Firenze Italy

**Keywords:** CCU, CO_2_ reduction, membranes, porous organic cage

## Abstract

Porous organic cages (POCs) are discrete molecular materials that combine intrinsic porosity with solution processability and well‐defined, chemically tunable cavities. These features make them attractive for CO_2_ capture and selective gas separation, and more recently for chemical transformations under confinement. Across amorphous and crystalline solids, as well as membrane and composite systems, POC‐based materials enable selective CO_2_ uptake. However, their performance is governed by how molecular structure translates into accessible pore environments through solid‐state organization. In catalysis, POCs can promote local CO_2_ enrichment and facilitate interaction with active sites, but their role extends beyond simple concentration effects. Even when not directly involved in the reaction, the POC's cavity can influence catalytic performance through confinement effects or host–guest interactions, as illustrated by hybrid systems and porphyrinic cages incorporating guest species. This Minireview examines recent advances in the use of POCs to integrate CO_2_ capture with confined reduction. We discuss how cage structure, cavity size, internal functionality, solid‐state packing, and processing strategies govern gas binding, transport, and accessibility, and how confinement influences catalytic behavior. These studies highlight emerging design principles but also current limitations, and point to clearer structure–function relationships to enable applications under realistic conditions.

## Graphical abstract

Porous organic cages (POCs) enable the coupling of CO_2_ capture and conversion through the interplay of molecular structure and solid‐state organization. Beyond intrinsic cavities, packing‐dependent pore accessibility and mass transport govern local CO_2_ concentration and catalytic performance, linking adsorption to reactivity in integrated capture–conversion systems.

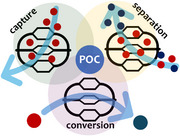

## 1 Introduction

The continuous rise in atmospheric CO_2_ concentration has intensified efforts to develop materials for technologies [1, 2] that transform carbon dioxide from an environmental issue into a chemical resource [3, 4, 5, 6, 7]. Beyond efficient capture, a key challenge is the design of systems that couple selective adsorption, transport, and catalytic conversion [8, 9, 10, 11, 12] within unified molecular environments capable of controlling substrate enrichment and reaction pathways. Achieving such integration [13, 14] requires porous platforms [15, 16, 17] featuring defined pore size and shape together with addressable internal functionalities [18].

Porous organic cages (POCs) [19, 20, 21, 22, 23, 24, 25, 26, 27, 28, 29, 30] represent a distinctive class of materials in this context. First reported by Cooper and co‐workers in 2009 [20], POCs are discrete, shape‐persistent molecules possessing permanent cavities that remain accessible in the solid state [23, 24, 25, 26, 27]. Unlike extended frameworks, their molecular nature affords intrinsically defined cavities with tunable functionality at both the cage interior and windows [20]. In the solid state, however, pore accessibility and transport are further modulated by intermolecular packing, while the discrete character of POCs preserves solution processability [22, 23, 24, 25, 26, 27, 28, 29, 30, 31, 32] and enables integration into extended frameworks [33] or hybrid architectures [34].

As a result, POCs have also emerged as promising platforms for the development of new porous materials for carbon management, ranging from post‐combustion CO_2_ capture and separation [35, 36, 37] to confined environments that can influence catalytic CO_2_ reduction pathways [38, 39, 40].

**FIGURE 1:**
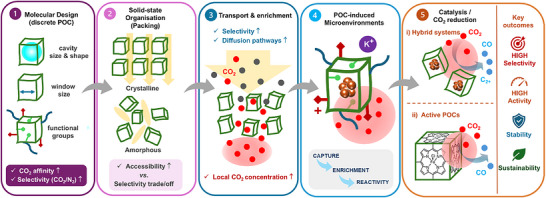
Multiscale control of CO_2_ capture, transport, and confined reduction in POC‐based systems.

In this Minireview, we discuss recent advances in POCs‐based materials, focusing on how control over molecular structure (cavity size, functionality) and solid‐state packing influence their performance in CO_2_‐selective sorption, separation, and catalytic reduction (see Figure 1). Owing to their porosity and structural tunability, POCs‐based systems can selectively capture CO_2_ while simultaneously generating confined microenvironments that promote local substrate enrichment and modulate mass transport. These features enable POCs to influence catalytic pathways, not only through direct incorporation of active sites, but also by controlling substrate concentration, stabilizing intermediates, and shaping the accessibility and reactivity of catalytic domains.

## 2 Structural Design of POCs for CO_2_ Capture

From the earliest studies on covalent organic cages as porous materials for carbon capture technologies, it became clear that supramolecular concepts, shared with host‐guest recognition, could play a central role. Maximizing interactions, indeed, requires the incorporation of multiple polar groups in the cage structure that can effectively engage with the electric quadrupole of CO_2_ [19, 20, 21, 22, 23, 41, 42]. Within this framework, moderately polar cage interiors defined, for example, by imine or imide functionalities [43, 44], P═O groups [45], or extended aromatic surfaces [46, 47] can promote favorable quadrupole–dipole interactions with CO_2_ while avoiding strong chemisorption. Following this strategy, most reported POCs exhibit isosteric heats of CO_2_ adsorption (Q_s__t_) in the range of 25–35 kJ mol^−1^ at zero coverage [23], providing an effective compromise between selectivity and reversible physisorption under ambient conditions.

At the same time, size exclusion emerged as a complementary design principle: early work converged on cage architectures with window apertures approaching the kinetic diameter of CO_2_ (ca. 0.33 nm), enabling selective diffusion and discrimination against larger and less polarizable gases, such as N_2_, relevant in post‐combustion applications [41, 48].

**FIGURE 2:**
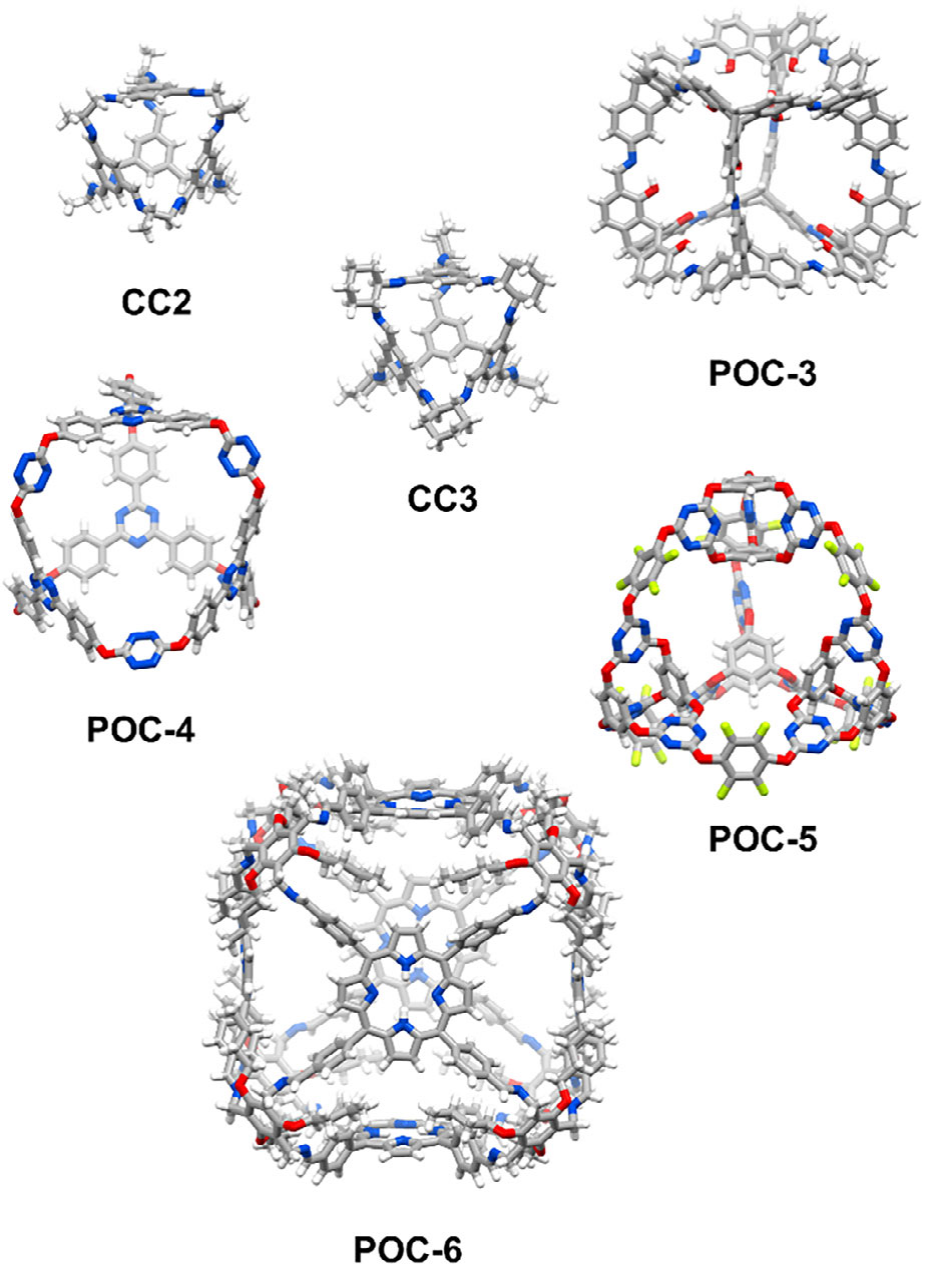
POCs reported in this Minireview. SCXRD data were obtained from the CCDC (CC2, CC3: 720849, 720850; POC‐3: 2270173; POC‐4: 2444420; POC‐5: 2303319; POC‐6: 1405313). Details on CO_2_ sorption results are summarized in Table S1.

CO_2_‐selective POCs are frequently synthesized using dynamic covalent chemistry (DCC) approaches, predominantly via imine condensation [19, 49, 50]. This modular synthetic route, supported by computational design [51, 52, 53] and high‐throughput technologies [53], enables systematic control over cage topology, cavity size, window aperture, and internal surface. Figure 2 shows the POC structures discussed in greater detail in this work (the corresponding CO_2_ adsorption and CO_2_/N_2_ selectivity data, summarized in Table S1).

Cooper's CCn cages [20], combining rigid imine‐based frameworks with narrowly defined apertures, have emerged as benchmark systems. In particular, crystalline CC3 and CC2 materials (Figure 2) effectively separate CO_2_ from N_2_ and H_2_ under ambient conditions (293 K, 1 bar). CC3 exhibits ideal adsorption selectivities of ∼8 for CO_2_/N_2_ and ∼20 for CO_2_/H_2_, while CC2 shows improved performance, with selectivities increasing to ∼9 and ∼35, respectively. This adsorption selectivity trend correlates linearly with gas polarizability ratios (e.g., CO_2_/N_2_ = 1.49; CO_2_/H_2_ = 3.21) [42, 54, 55, 56].

However, in intrinsically porous molecular materials such as POCs, gas sorption is strongly influenced by molecular packing in the solid state [36]. Packing generates extrinsic porosity, which in turn governs access to the intrinsic cage cavities. This effect, already noted in earlier studies [18, 19, 20, 21, 22, 23], has been more recently examined by Mastalerz et al., who showed that crystalline POCs generally exhibit higher surface areas and gas uptakes, albeit at the expense of CO_2_/N_2_ selectivity. For example, crystalline and amorphous forms of POC‐3 display maximum CO_2_ uptakes of 5.01 and 4.10 mmol g^−1^ at 273 K and 1 bar, respectively, while the corresponding CO_2_/N_2_ Henry's selectivities at zero coverage are ∼14 for the crystalline phase and ∼35 for the amorphous one (see Tables S1) [57]. Controlling packing modes and polymorphism is therefore crucial, although the weak and adaptive nature of intermolecular interactions makes rational design challenging [58]. To address this limitation, cooperative multiscale assembly strategies [59], for example, those based on double‐solvent systems or ionic surfactants, have proven effective in directing solid‐state organization. These approaches enable the formation of hierarchically ordered POC‐based materials [60, 61], including macroscopic crystals with aligned pore channels, one‐dimensional microtubes, and two‐ or three‐dimensional porous superstructures. Such porous architectures can also accommodate catalysts, offering a platform for integrating capture and reactivity. This, in turn, opens up opportunities to develop selective POCs materials that effectively combine CO_2_ capture with subsequent conversion processes.

The translation of POCs into industrial technologies related to carbon capture remains, however, constrained by several challenges: (i) scalable syntheses of high‐performing porous cages [62] and production costs; (ii) chemical stability under moist and acidic flue‐gas conditions [63, 64, 65, 66]; (iii) competitive water adsorption [67, 68]. Addressing the first challenge, Li and Ma demonstrated in 2023 that high‐pressure homogenization (HPH) enables large‐scale production of high‐quality crystalline POCs, achieving production rates of up to 2.1 × 10^4^ kg m^−3^ day^−1^ with solvent recycling [62]. From a practical general perspective, production cost considerations further highlight the potential of organic cages compared to other porous materials. In particular, in contrast to many MOFs [16], whose synthesis often relies on metal salts, high‐boiling solvents (e.g., DMF), and energy‐intensive solvothermal conditions followed by activation steps, POCs are typically assembled from purely organic precursors via comparatively mild solution‐phase reactions. This can reduce both economic costs and environmental burdens associated with metal sourcing, processing, and end‐of‐life recovery. Although comprehensive techno‐economic analyses remain limited [16], these factors suggest a potential advantage of POCs in developing scalable and sustainable CO_2_ capture and utilization technologies.

Chemical stability remains a central issue, as the intrinsic reversibility of DCC can compromise long‐term robustness under humid and acidic conditions (e.g., SO_2_‐ and H_2_S‐containing streams) [69, 70]. This limitation affects not only post‐combustion carbon capture, but also the implementation of integrated CO_2_ capture–reduction processes, where durable performance under reactive conditions is essential.

To address this issue, a range of post‐synthetic stabilization strategies has been developed over the years [71], including imine reduction, [3,3]diaza‐Cope rearrangement [72], Povarov cyclization [65]. Reduction to amines generally increases the structural flexibility of POCs and often reduces their permanent porosity [19], thereby motivating the development of more rigid derivatives via the subsequent conversion of amine linkages into amides [66, 73, 74], carbamates [75], and cyclic ureas [76]. In the most successful cases, such as the carbamate cage reported by Mastalerz [75], this transformation preserves CO_2_ uptake (∼2.6 mmol g^−1^ at 273 K and 1 bar) while markedly enhancing stability under strongly acidic (1 M HCl) and basic (1 M NaOH) conditions. Chemically robust POCs have also been accessed through irreversible bond‐forming reactions, including nucleophilic aromatic substitution (SN_Ar_) [19, 77, 78, 79]. In a recent example, Liu and co‐workers reported the tetrazine‐based **POC‐4** (Figure 2) that combines high surface area (>1000 m^2^ g^−1^) with CO_2_‐selective adsorption (4.2 mmol g^−1^ at 273 K, 1 bar), together with remarkable stability in boiling water and under strongly acidic and basic conditions [80]. Importantly, the use of chemically stable POCs as building blocks for cage‐containing frameworks offers a promising route to novel, robust porous materials with enhanced CO_2_ uptake for real separation applications. For instance, in 2024, Cooper et al. employed a non‐porous POC as a building block to construct a very large [4[2+3]+6] cage (see **POC‐5**, Figure 2) with excellent hydrolytic stability and CO_2_ uptake (∼4.0 mmol g^−1^ at 273 K, 1 bar) [81].

A further challenge for practical applications is mitigating competitive water adsorption, as water typically binds more strongly than nonpolar CO_2_ in porous materials [67, 68]. In 2025, Cooper and coworkers [67] demonstrated through bottom‐up computational screening that shape‐persistent, ultra‐hydrophobic macrocycles (0.7–0.8 nm) can stabilize CO_2_ via the cooperative action of multiple π─π interactions, collectively overcoming the dipole‐driven interactions responsible for water binding. Experimental validation on macrocycles confirmed preserved CO_2_ uptake under humid conditions, outperforming benchmark metal‐organic frameworks and zeolites at >75% relative humidity. These findings suggest that analogous design principles may be extended to future POCs.

**FIGURE 3:**
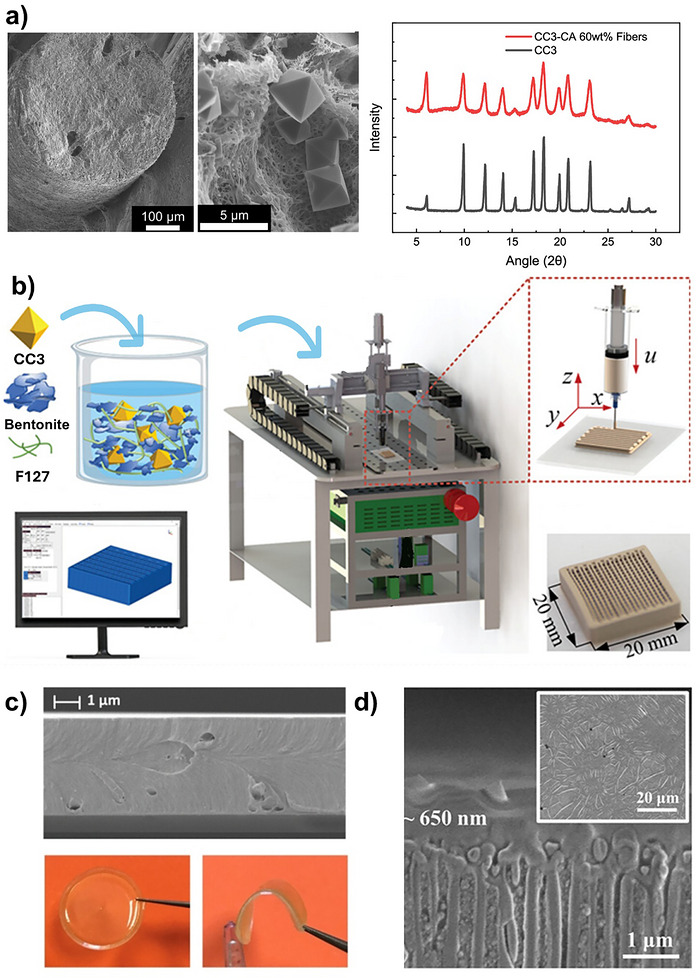
(a) left: SEM images of **CC3**–cellulose acetate (**CC3**–CA) fibers (left) and PXRD patterns of **CC3**–CA fibers compared with the reference **CC3** compound (right). Adapted with permission from Ref. [83] 2021 American Chemical Society. (b) Schematic illustration of the 3D‐printing process. Left: printable inks composed of **CC3**, bentonite, Pluronic F‐127, ethanol, and water. Centre and right: direct ink writing and subsequent drying steps used to reproduce the computer‐designed structures. Adapted from Ref. [82], under CC BY license 2024 The Authors, published by Wiley‐VCH GmbH. (c) Self‐standing, mechanically robust, dense films of a neat POC. Adapted from Ref. [84] under CC BY license 2023 The Authors. published by Wiley‐VCH GmbH. (d) FESEM images of recrystallized BMIMBF_4_@**CC3** membrane. Adapted with permission from Ref. [85] 2022 Wiley‐VCH GmbH.

For implementation in gas separation technologies, POCs must typically be processed from powders into structured forms such as pellets, films, or membranes (see Figure 3) [82, 83]. The direct use of solid sorbents in powder form indeed presents practical limitations, including handling. Immobilization within solid architectures is therefore required to enable integration into devices (e.g., filters) without compromising adsorption performance. To this end, a range of fabrication strategies has been developed, including extrusion‐based methods and 3D printing via direct ink writing (DIW), which enable the formation of fibers and three‐dimensional architectures while preserving the intrinsic cage structure and gas sorption properties. Beyond gas capture and separation, the development of hierarchically porous POC‐based architectures opens opportunities for integrated capture–conversion systems. The combination of nanometer‐scale cavities, tunable micro‐ and mesoporosity within printed filaments, and macroscopic flow channels enables control over gas transport, residence time, and local CO_2_ concentration. These features are particularly relevant for catalytic applications, where POCs can function both as selective adsorbents and as supports for dispersed catalytic species, enhancing reactivity, stability, and recyclability. In this context, structuring POCs into three‐dimensional architectures may facilitate CO_2_ capture coupled with confined reduction processes by improving mass transport and promoting efficient interaction between adsorbed CO_2_ and catalytic sites. Such multiscale design strategies provide a promising platform for bridging capture and reactivity in process‐relevant materials.

## 3 POCs‐Derived Materials and Membranes

The previous section of this work focused on the structural features of discrete POCs that govern their affinity and selectivity toward CO_2_, in relation to their use as adsorbents for carbon capture. However, POCs‐based materials development in this area extends beyond adsorption to include CO_2_ separation through membranes. These approaches rely on different mechanisms (adsorption by solid sorbents vs. permeation through membranes), but share key requirements such as selectivity and chemical stability of the materials employed. These aspects become particularly important in the context of integrated capture‐conversion systems, where materials must remain stable under reaction conditions and enable controlled mass transport of CO_2_ and intermediates.

**FIGURE 4:**
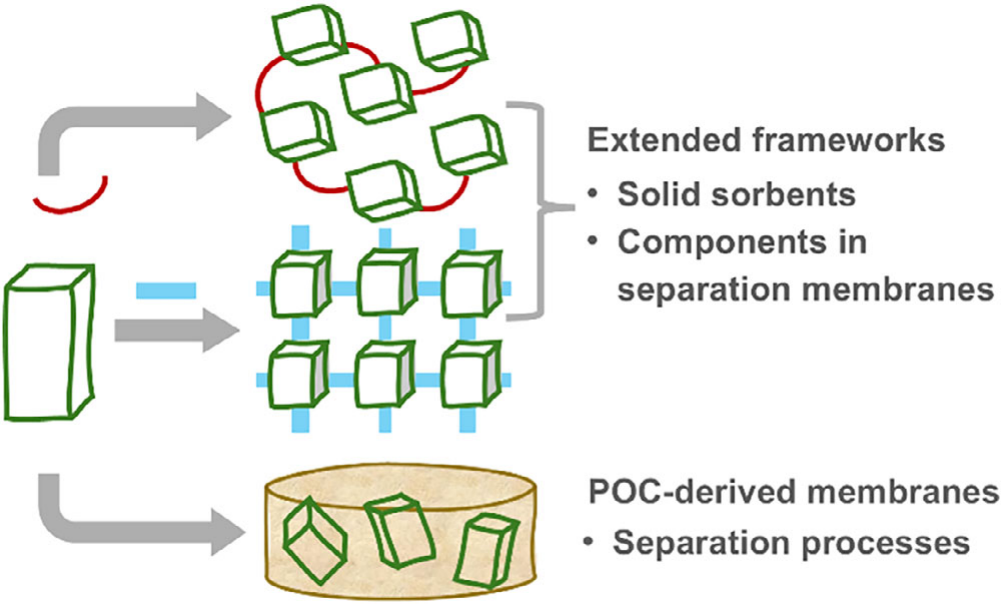
POC‐derived extended frameworks, spanning ordered (3D COF‐like) and disordered polymer‐like architectures, as well as membranes (e.g., MMMs, where POCs are embedded in an organic polymer matrix).

POCs have also been used as building blocks in extended organic frameworks (Figure 4) [33, 86, 87, 88]. These materials remain closely connected to the parent cages, as their intrinsic cavities, chemical functionality, and host‐guest interactions can be partially preserved or translated into the extended structure. In such systems, the intrinsic porosity of the cage is retained, while the resulting materials benefit from the robustness of porous organic polymers or covalent organic frameworks and can exhibit enhanced adsorption properties compared to the corresponding discrete cages, due to increased accessibility and modified pore environments. However, this structural translation is not always straightforward: partial loss of cavity definition, reduced accessibility, or the emergence of non‐selective interstitial porosity can limit the direct transfer of molecular‐level selectivity to the bulk material.

In this section, we describe how POCs have been employed to construct robust, CO_2_‐selective extended frameworks, and how POC‐based materials (either composed of discrete units or cross‐linked networks) can be processed into membranes for CO_2_ separation from gas mixtures (Figure 4) [28, 31, 35, 50, 84, 89, 90, 91].

### 3.1 Cage‐Derived Extended Organic Frameworks

Cage‐to‐framework strategies provide a route to stabilize POCs and tune pore accessibility, with direct consequences for CO_2_ adsorption and selectivity. One of the earliest examples was reported by W. Zhang and co‐workers in 2011. However, despite the high CO_2_/N_2_ ideal adsorption selectivity (up to 213) and good chemical stability, these materials exhibited a relatively low CO_2_ uptake (0.16 mmol g^−1^ at 273 K and 1 bar) [92, 93].

A few years later, C. Zhang and co‐workers demonstrated that incorporation of tetraphenylethylene‐based oxacalixarene cages [94] into porous organic polymer frameworks enhances the adsorption performance of the parent cage. Polymerization disrupts the window‐to‐arene packing motif of the discrete cages, thereby activating intrinsic cavities and increasing accessible porosity. As a result, the polymer exhibits higher surface area and pore volume, with CO_2_ uptake increasing from 0.60 to 2.20 mmol g^−1^ at 273 K (1 bar) and CO_2_/N_2_ selectivity from 9 to 27. The replacement of tetraphenylethylene units with electron‐deficient pyrazine [95] or triazine [96] motifs further strengthen CO_2_ binding through dipole‐quadrupole interactions, leading to uptakes of up to 2.69 mmol g^−1^ (vs. 0.54 mmol g^−1^ for the precursor cage at 273 K, 1 bar). Improved CO_2_/N_2_ selectivity relative to the corresponding discrete cage was also achieved during the polymerization of tetrazine‐based POC‐4 [80].

Cage‐derived 2D and 3D COFs with significantly improved porosity with respect to the discrete cage components have also been reported. For instance, the 2D COF reported by Whang et al. exhibits pronounced CO_2_ affinity, with an uptake capacity of up to 1.95 mmol g^−1^ at 273 K (1 bar) [96]. Building on this example, Cooper et al. synthesized a crystalline 3D COF from a non‐porous hexa‐amino POC and 2,5‐dihydroxyterephthalaldehyde, achieving CO_2_ uptakes of up to 4.6 mmol g^−1^ at 273 K (1 bar) [97, 98].

POCs have also been successfully employed as building units for amorphous porous organic polymers [99, 100, 101]. In the examples by Coskun et al., the resulting porous polymers reached CO_2_ uptakes of up to 4.21 mmol g^−1^ at 273 K and 1 bar, along with CO_2_/N_2_ IAST selectivities [100, 101] of up to 100. The relatively high isosteric heats of adsorption measured for these polymers with CO_2_ (Q_s__t_ up to ∼43 kJ mol^−1^) are attributed to strong host–guest interactions within the ultramicroporous cavities.

The broad compatibility of POCs with diverse monomers enables the construction of porous architectures with well‐defined pore environments and connectivity. These features highlight how cage‐derived materials can translate molecular‐level design into tunable adsorption and separation performance, and make them promising candidates for advanced gas separation applications, including membrane‐based systems.

### 3.2 POC‐Based Membranes for CO_2_ Separation

Membrane‐based separation is a key technology for CO_2_ capture from gas mixtures, with applications ranging from natural gas and biogas upgrading to post‐combustion streams [102, 103, 104, 105, 106]. Compared to energy‐intensive processes such as cryogenic distillation or pressure swing adsorption, membranes offer a more energy‐efficient alternative. However, achieving both high CO_2_ permeability and selectivity in conventional polymer membranes remains challenging due to the well‐known Robeson trade‐off [107, 108].

**FIGURE 5:**
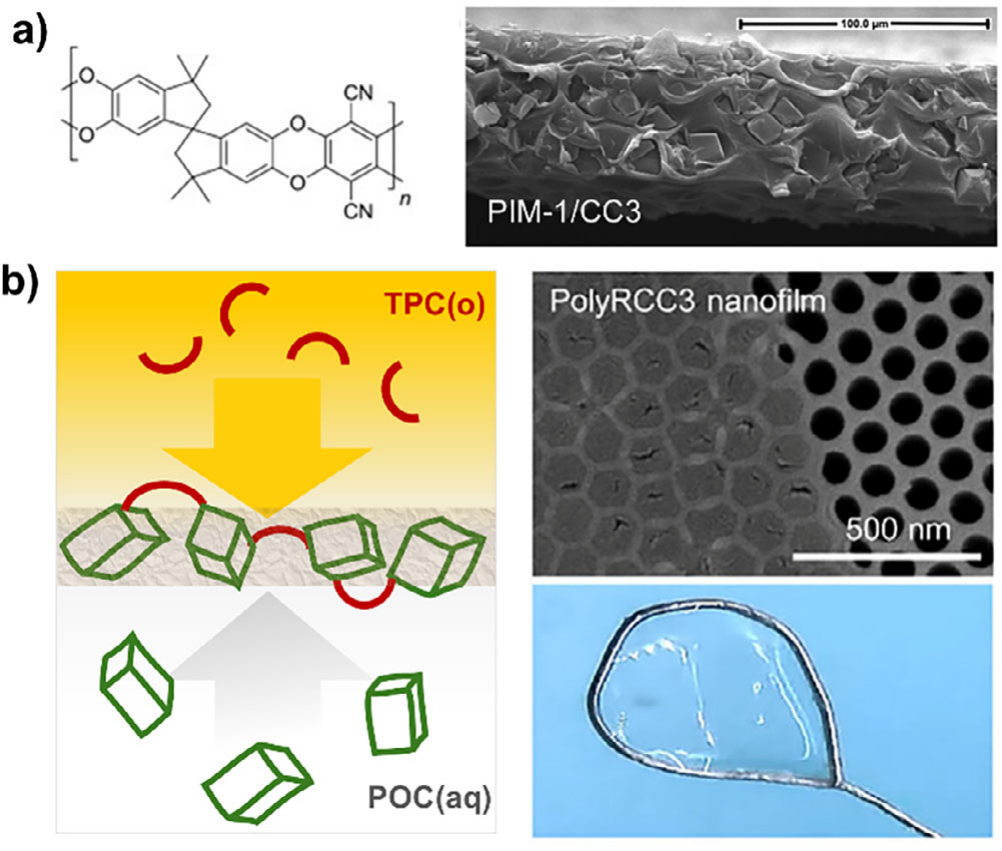
(a) SEM image of a PIM‐1/**CC3** MMM (10:2 wt ratio, image of the cross section). Adapted with permission from Ref. [113] 2013 Wiley‐VCH Verlag GmbH & Co. KGaA, Weinheim. (b) left: Schematized interfacial reaction between POC (e.g. **RCC2**, **RCC3**, and derivatives) and TPC; right: picture and SEM image of polycage **RCC3** nanofilm transferred onto an anodized aluminum oxide (AAO) support. Adapted with permission from Ref. [115] 2026 Wiley‐VCH GmbH.

In this context, the incorporation of discrete porous molecules, such as POCs, into membrane materials has emerged as an effective strategy to enhance CO_2_ separation performance [35]. POCs introduce selective sorption sites, disrupt polymer chain packing, and generate additional free volume, thereby promoting preferential transport pathways for CO_2_ [106]. A straightforward approach involves dispersing POCs within polymer matrices to form mixed‐matrix membranes (MMMs) [35, 109, 110, 111, 112]. An early example is the in‐situ crystallization of CC3 within a PIM‐1 matrix, yielding MMMs (Figure 5a) with enhanced CO_2_ permeability while maintaining selectivity and improving resistance to physical ageing. A relevant increase in CO_2_ permeance can be achieved by blending either pure POCs [113] or mixtures of scrambled POCs with polymer matrices, as demonstrated by Lively and co‐workers using scrambled amorphous cages [114].

Continuous membranes composed entirely of POCs have also been developed. In 2016, Cooper et al. prepared crystalline cage membranes by spin‐coating CC3 onto porous anodic alumina supports [116, 117], achieving CO_2_ permeances of up to 2746 GPU and a CO_2_/N_2_ ideal selectivity of 18.7 at 1 bar. Defects arising from disordered CC3 packing can be mitigated by introducing additives, such as ionic liquids, during membrane formation [104, 105]. In particular, Xu and co‐workers showed that electrostatic interactions between 1‐butyl‐3‐methylimidazolium tetrafluoroborate (BMIMBF_4_, Figure 3d) and CC3 lead to defect‐free crystalline membranes with enhanced CO_2_/N_2_ selectivity (>130), albeit at the expense of reduced CO_2_ permeance [85].

**FIGURE 6:**
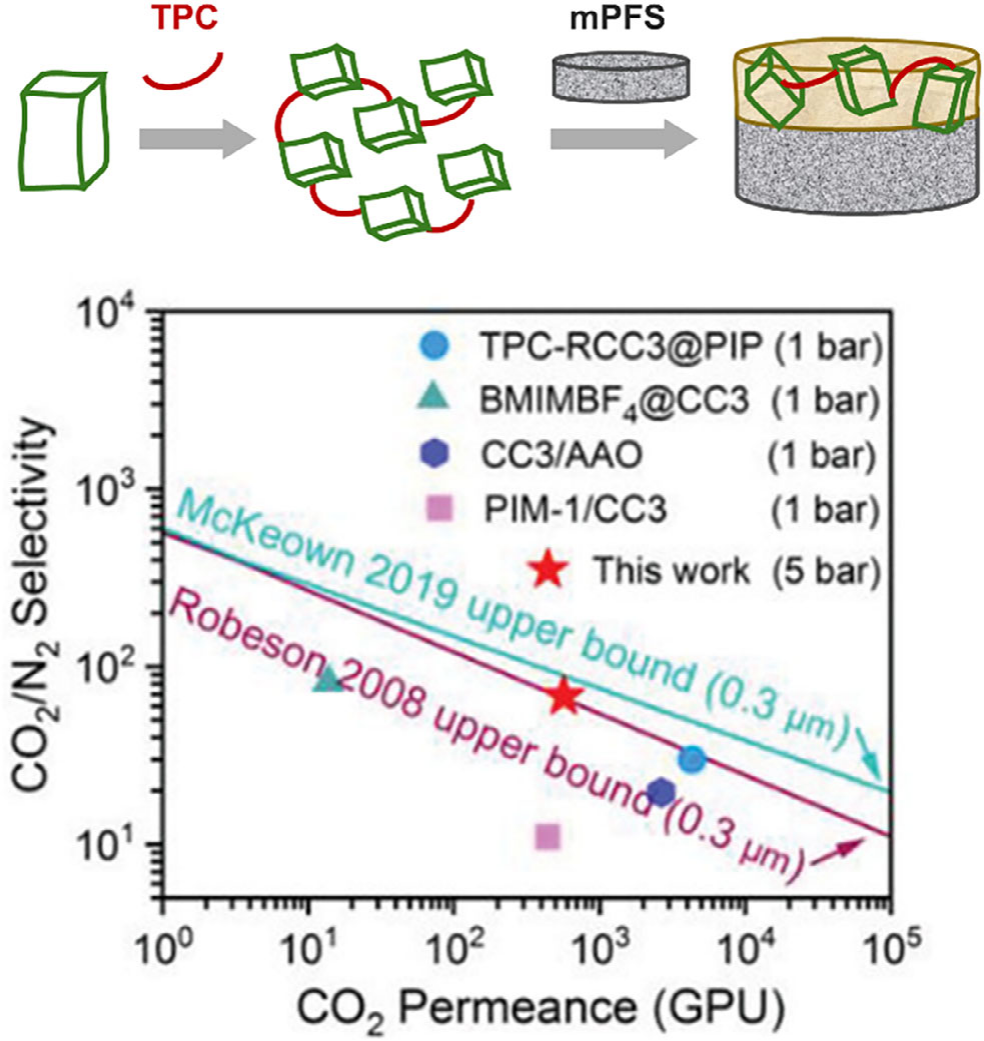
POC–TPC/mPSf composite membrane. The membrane is fabricated via interfacial reaction between the POC and TPC on a PSf support (mPSf) pre‐coated with a polydimethylsiloxane (PDMS) layer. Bottom: gas permeance and selectivity of the POC–TPC/mPSf composite membrane (red stars) compared with literature‐reported POC‐based systems, see Ref.s [113] and [85]. Adapted with permission from Ref. [119] 2024 Wiley‐VCH GmbH.

An alternative route to defect‐free membranes for CO_2_/N_2_ separation involves the interfacial polymerization of POCs (Figure 5b) on porous supports. For instance, composite membranes can be fabricated via interfacial reaction of reduced CC3 (RCC3) with terephthaloyl chloride (TPC) on modified polysulfone (mPSf) supports. These membranes, reported by Zhao and coworkers, exhibit CO_2_ permeances of up to 4303 GPU and CO_2_/N_2_ selectivities of 30 at 1 bar (Figure 6) [118]. Further improvements, with selectivity up to 42.4, were achieved using ultrathin polycage layers derived from water‐soluble amine‐rich RCC3 derivatives [115]. Importantly, this approach is compatible with large‐scale fabrication: composite membranes based on reduced CC2 and TPC have been produced over areas up to 1200 m^2^, while maintaining high performance (CO_2_ permeance of 1141 GPU and CO_2_/N_2_ selectivity of 51 at 1 bar) [119]. The combination of scalability, high‐pressure stability, and high CO_2_ purity under simulated flue‐gas conditions (up to 99.5%) highlights the potential of polycage membranes for practical CO_2_ separation. Taken together, these studies show how POC‐based materials enable control over CO_2_ uptake and transport across multiple length scales. Nevertheless, balancing permeability, selectivity, and long‐term stability remains a key challenge, particularly under realistic operating conditions. However, beyond separation, such control over confinement and local concentration provides a basis for coupling selective CO_2_ separation with its subsequent transformation.

## 4 POCs as Microenvironments for CO_2_ Reduction

Building on their ability to control CO_2_ uptake and transport, POC‐based materials can influence reactivity in confined environments, particularly in CO_2_ reduction. Here, “confinement” does not necessarily imply that CO_2_ reduction occurs within the intrinsic cavity of the cage; rather, it refers more broadly to the local chemical environment created by POCs at or near catalytic interfaces, which modulates CO_2_ concentration, mass transport, and intermediate stabilization. Electrochemical [13, 120], photocatalytic [121, 122, 123, 124], and photoelectrochemical [125, 126] pathways share common requirements, including efficient mass transport, stabilization of reactive intermediates, and controlled substrate availability at catalytic interfaces. From a technological perspective, electrolysis‐based CO_2_ conversion [127, 128] is currently the most advanced route toward industrial implementation, particularly for the production of CO, syngas [129], methane, and formate [130], in flow‐cell and solid oxide systems, where selectivity, durability, and transport phenomena determine overall performance. On the other hand, photocatalytic and photoelectrochemical approaches remain less developed, largely due to challenges in photon management and scalability [123].

Rather than acting as primary catalytic sites, POCs more often function as microenvironments that influence catalytic performance through confinement, host‐guest interactions, and mass transport. By enriching CO_2_ near active sites and regulating access to reactive domains, they can alter reaction pathways, enhance selectivity, and promote multi‐electron transformations [125, 126]. Importantly, these effects go beyond a purely structural or support role, as confinement within POC‐derived environments can directly influence catalytic pathways and product distribution. Accordingly, POCs can contribute to catalysis [131, 132, 133, 134] either indirectly, as modifiers of the local environment, or directly, when catalytically active functionalities are incorporated within the cage structure. In most reported systems, this effect is predominantly interfacial, whereas true intracavity catalysis is difficult to demonstrate unambiguously.

### 4.1 Hybrid Systems: POCs With Metal Nanoparticles

The intrinsic cavities of POCs provide spatially confined domains capable of stabilizing metal catalysts, such as clusters and nanoparticles (MNPs; Cu, Pd, Pt, Au, Rh) [135]. Confinement of MNPs within POC cavities offers several advantages: it enables controlled nucleation, leading to narrowly distributed nanoparticles, and suppresses high‐surface‐energy‐driven aggregation. The resulting hybrid assemblies are attractive for their ability to bridge homogeneous and heterogeneous catalysis. However, confinement of MNPs within POCs’ cavities can limit substrate diffusion due to partial or complete pore occupation [136, 137, 138].

Li and co‐workers addressed this limitation by positioning catalytically active MNPs within the extrinsic voids of POC‐based materials. In this configuration, sorption capacity and mass transport are preserved, while POCs enhance catalytic performance by generating a substrate‐enriched microenvironment near the metal active sites. Hierarchically organized POC‐derived architectures expand this diffusion‐engineering strategy [59, 60, 87]. In particular, CC3‐based microtube arrays spatially compartmentalize Pd clusters and enzymes within distinct pore domains, thus enabling tandem chemoenzymatic transformations [59]. Although demonstrated in organic synthesis [136], this approach provides a conceptual foundation for integrated CO_2_ capture–reduction platforms.

**FIGURE 7:**
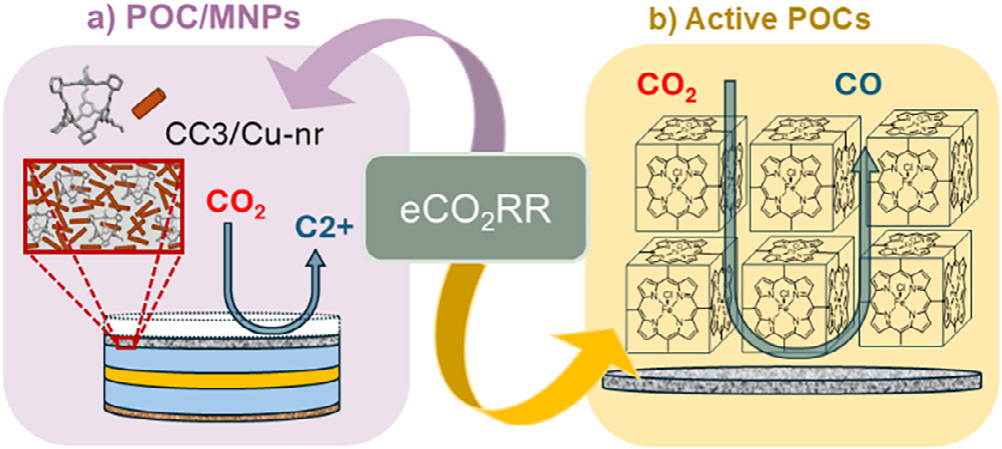
Applications of POCs in eCO_2_RR. In strategy (a), the POC increases the local CO_2_ concentration at the active sites (MNPs); in strategy (b), the POC itself acts as a selective catalyst for CO_2_ reduction. See details on the reported systems in Tables S2 and S3.

**FIGURE 8:**
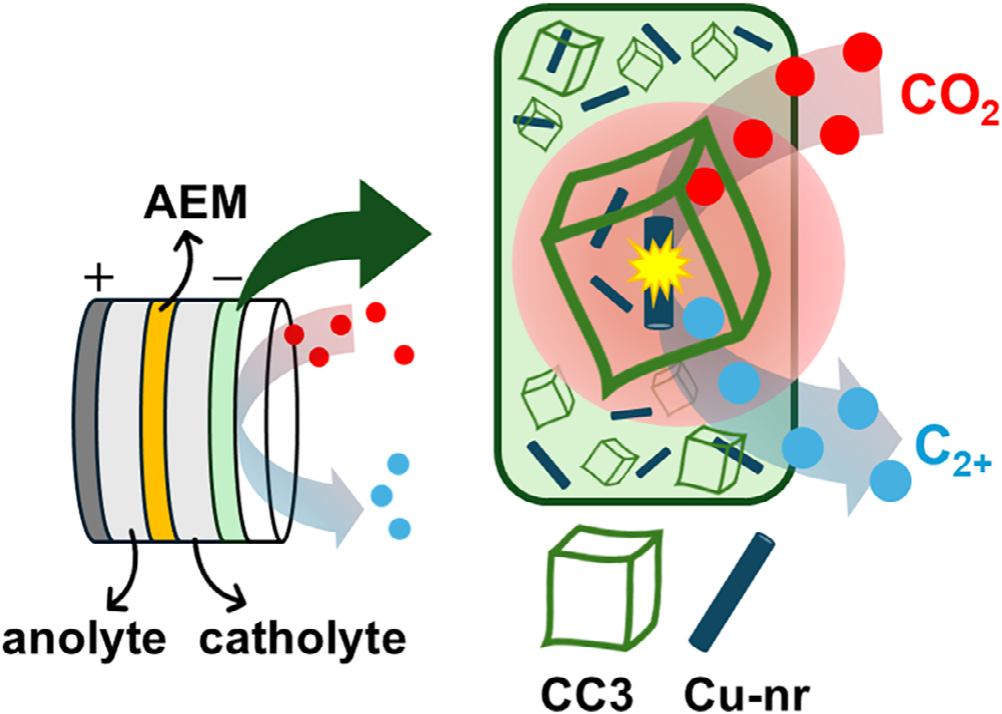
Schematic representation of the flow‐cell configuration for the electrolytic reduction of CO_2_. An anion‐exchange membrane (AEM) enables OH^−^ transport from the cathode (‐, GDE) to the anode (+). CC3 can be integrated within the catalyst layer of GDEs where it acts as CO_2_‐enriching microenvironments without compromising gas transport through the GDL [139].

Electrochemical CO_2_ reduction (CO_2_RR) offers a promising route for converting CO_2_ into fuels and value‐added chemicals using renewable electricity. Recent advances in flow‐cell configurations [137, 138] employing catalysts deposited on gas‐diffusion electrodes (GDEs) have markedly enhanced CO_2_RR performance, enabling operation at industrially relevant current densities [15]. In 2022, Han and coworkers [139] demonstrated that POCs hybridized with MNPs can enhance CO_2_ flux toward the electrode by locally enriching CO_2_ concentration at the catalyst–electrolyte interface (Figure 7). In particular, CC3 was combined with CuO nanorods (Cu‐nr) [140, 141], and the resulting hybrid catalyst was deposited onto hydrophobic PTFE gas‐diffusion layers (GDLs), see Figure 8. In this configuration, CC3 acts as a local CO_2_ reservoir, promoting faster transport of CO_2_ to Cu‐nr than is possible through diffusion in the electrolyte alone. Using this Cu‐nr/CC3 assembly, a Faradaic efficiency (FE) of 76.1% for C_2+_ products was achieved at 1.7 A cm^−2^. This strategy also proved to be generalizable: Ag/CC3 reached 95.3% FE for CO at 0.39 A cm^−2^, compared to 85.8% and 0.16 A cm^−2^ without CC3, while Bi_2_O_3_/CC3 delivered 77.1% FE for HCOO^−^ at 0.28 A cm^−2^, versus 51.0% and 0.14 A cm^−2^ for the bare catalyst (see also Table S2). These comparisons highlight that the presence of POCs not only increases CO_2_ availability but also leads to measurable improvements in catalytic performance metrics, including current density and product selectivity, consistent with a microenvironment‐driven effect rather than a passive support function (see the Supporting Information).

**FIGURE 9:**
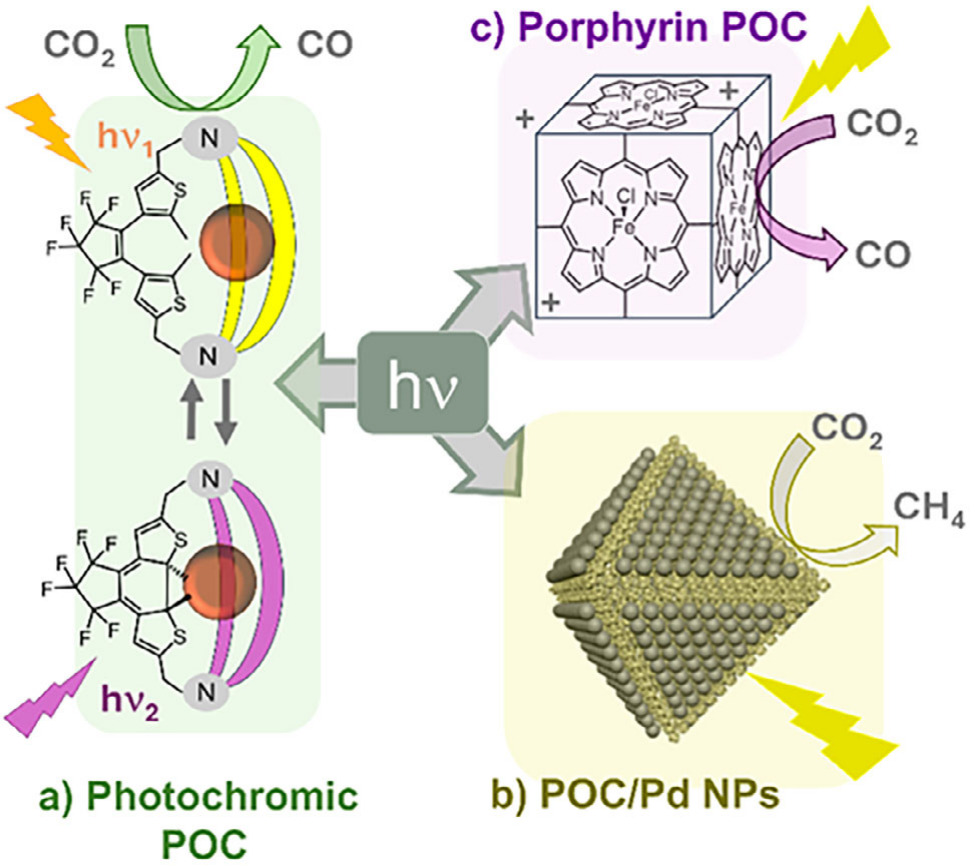
Selected examples of POCs in photocatalytic CO_2_ reduction: (a) CO formation with an Au/DTE‐POC hybrid system featuring Au NPs confined within a photochromic dithienylethene (DTE) cage [142]; (b) CH_4_ production from CO_2_ using Pd NPs co‐assembled with CC3 and [Ru(bpy)_3_]^2+^ as photosensitizer. Image adapted from Ref. [143] with permission from Elsevier, 2023 Elsevier; (c) CO generation using a cationic porphyrinic POC in combination with Ir(ppy)_3_. The performance of the reported POC‐based systems are summarized in Tables S2 and S3.

Beyond electrocatalysis, POC/MNP hybrids have also been explored as platforms for the photocatalytic reduction of CO_2_ (Figure 9). In 2021, Maji and coworkers reported an Au/DTE‐POC hybrid system obtained by incorporating ultrasmall (<2 nm) Au nanoparticles into a photochromic dithienylethene cage (DTE‐POC) [142].

The resulting metal‐organic interface forms an Au/DTE‐POC Schottky junction, which enhances charge separation under visible irradiation (400‐750 nm), as evidenced by a ∼2.5‐fold increase in photocurrent compared to the pristine POC. The Au/DTE‐POC hybrid catalyses the CO_2_‐to‐CO photoreduction, producing 617 µmol of CO under visible‐light irradiation, increasing to 1178 µmol under broader spectral excitation (see also Table S2).

A distinct paradigm is represented by Pd nanoparticles (∼4.7 nm) co‐assembled with CC3 in the presence of [Ru(bpy)_3_]^2+^ and a sacrificial donor [143]. Here, photogenerated electrons are transferred from the excited Ru complex to Pd NPs, while the cage functions as a CO_2_‐enriching microenvironment. Bare Pd NPs exhibit high selectivity (∼97%) but modest activity (∼ 10 µmol g^−1^ h^−1^) in CH_4_ formation. Integration with CC3 increases the CH_4_ formation rate to 78.5 µmol g^−1^ h^−1^ while maintaining ∼98% selectivity. This indicates that increasing CO_2_ concentration facilitates the demanding eight‐electron CO_2_‐to‐CH_4_ reduction pathway. This result further illustrates how local CO_2_ enrichment within the cage environment can facilitate demanding multi‐electron transformations that are otherwise kinetically limited.

Collectively, these electrochemical and photocatalytic studies highlight two design approaches for CO_2_ reduction based on hybrid POC/MNP systems: (i) electronic‐interface engineering, relying on confinement of MNPs to promote charge separation, and (ii) microenvironment engineering, which focuses on tuning the local chemical environment around active sites. Overall, these systems demonstrate that POCs actively modulate the catalytic environment by controlling local CO_2_ concentration, mass transport, and interfacial processes, thereby influencing activity and selectivity beyond a simple structural role.

### 4.2 Catalytically Active POCs

**FIGURE 10:**
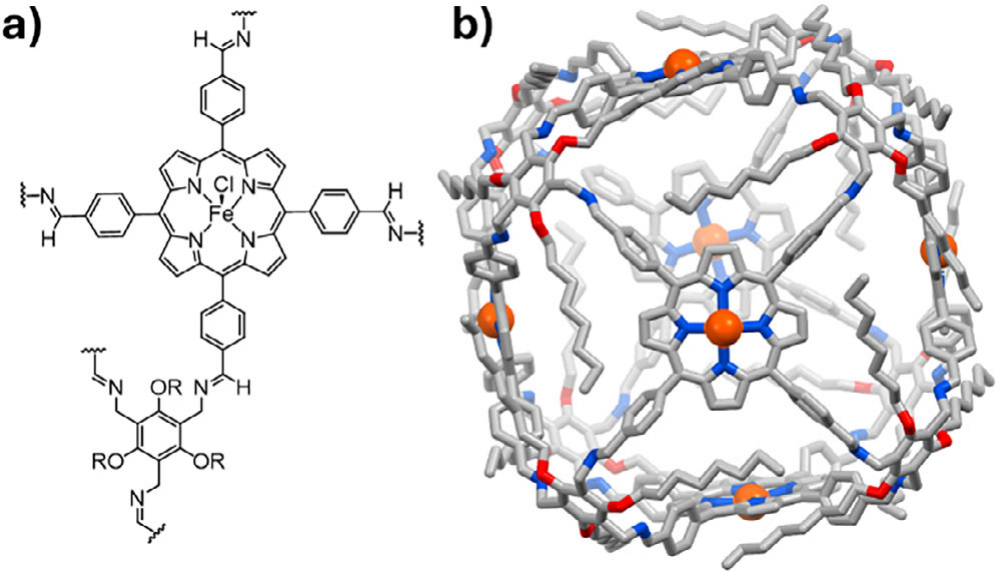
(a) Representative portion of the FePB cage reported by Kim, Chang et al. in Ref. [144], R═CH_3_(CH_2_)_7_‐; (b) SCXRD structure of the zinc analogue complex of FePB taken from CCDC (CCDC 1544697) [146].

In the systems discussed above, POCs primarily influence catalysis indirectly through microenvironmental effects; however, a complementary strategy is to embed catalytically active sites directly within the cage structure, thereby enabling a more direct role in CO_2_ reduction. Efficient CO_2_ reduction catalysts have been developed by integrating metalloporphyrins or metallophthalocyanines into POC structures. Upon incorporation of catalysts such as iron(III) tetraphenylporphyrin (FeTPP), the resulting systems exhibit high selectivity for CO_2_‐to‐CO conversion (see Table S3). Moreover, the cage framework increases the local CO_2_ concentration near the active centres while maintaining their spatial separation, thereby suppressing porphyrin aggregation. In a seminal study, Kim, Chang and coworkers reported a rhombicuboctahedral cage (FePB, Figure 10) containing six FeTPP units [144], which showed enhanced CO‐specific current densities compared to FeTPP, particularly under mass transport‐limited conditions. This enhancement highlights the role of the cage architecture in improving CO_2_ accessibility and local concentration at catalytically active sites [144, 145, 146, 147].

Further improvements were achieved using cationic porphyrinic POCs [148]. Controlled potential electrolysis afforded CO with high FE (∼80%), while photocatalytic experiments reached 97% selectivity, TON = 7006, and TOF = 1429 min^−1^. These results indicate that confinement combined with electrostatic effects can significantly enhance both activity and selectivity, even at low CO_2_ concentrations. Performance results are summarized in Table S3.

Host–guest binding offers an additional strategy to modulate catalytic performance. A representative example is provided by oxyethylene‐functionalized porphyrin cages, which can complex K^+^ ions through the oxyethylene groups, generating a local electrostatic environment that stabilizes key intermediates and lowers overpotentials, thereby enhancing catalytic activity [135]. Cofacial porphyrinic cage architectures further improve performance: the enforced spatial organization of active sites leads to higher activity compared to monomeric analogues and enables CO Faradaic efficiencies approaching 90% [149]. Along similar lines, encapsulation of fullerene guests, particularly C_70_, within the cage cavity represents another manifestation of host–guest effects, enhancing catalytic performance most likely by increasing structural rigidity rather than through direct electronic contributions [150].

**FIGURE 11:**
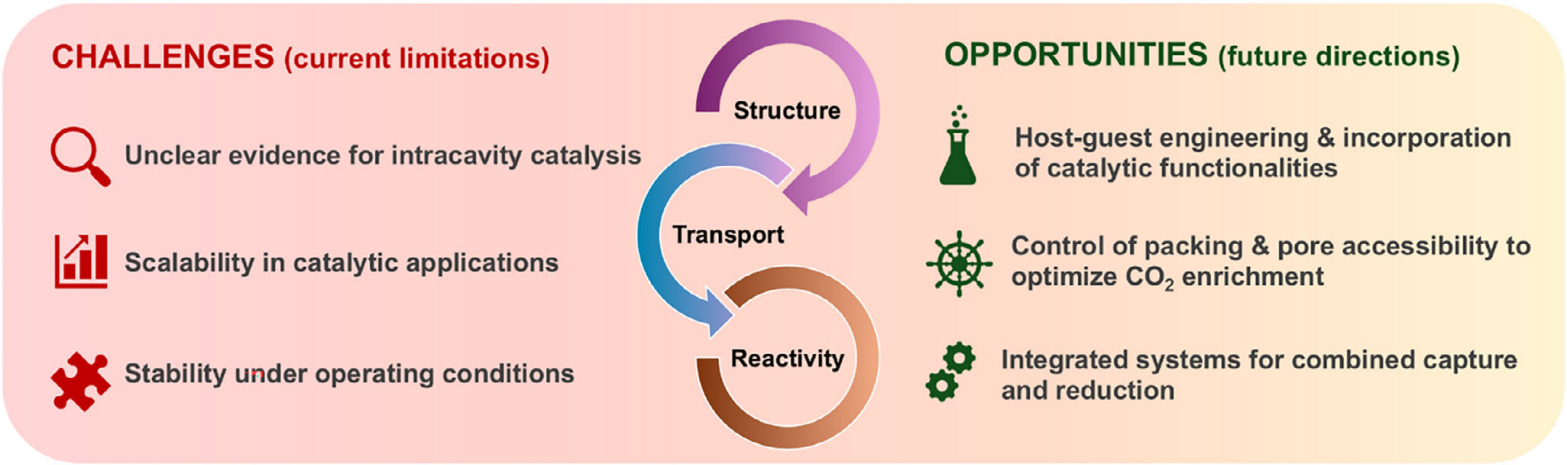
Current limitations and future opportunities for the application of POCs‐based materials in combined CO_2_ capture and reduction processes.

Despite these advances, several limitations remain (Figure 11). In many systems, the respective contributions of local CO_2_ concentration, mass transport, and intrinsic catalytic activity are difficult to disentangle, and clear evidence for true intracavity catalysis is still limited. In addition, stability under operating conditions and scalability of POC‐based systems remain important challenges.

## 5 Summary and outlook

A recurrent observation across these studies is that the performance of CO_2_‐selective POCs‐based materials depends not only on the cage structure, but also on how this structure translates into accessible pore environments in the solid state. Solid‐state organization strongly influences mass transport and the accessibility of active sites and can, in some cases, outweigh the effects of molecular design.

POCs exhibit promising performance as CO_2_ capture sorbents, with selectivity and sorption capacity (Table S1) comparable to benchmark MOFs [16]. Additionally, they mitigate costs and environmental impacts related to metal sourcing, processing, and disposal, while their higher solubility compared to MOFs and other frameworks allows for more straightforward solution processing.

When applied in the reduction of CO_2_, POCs generate confined microenvironments that locally increase CO_2_ concentration and promote its interaction with catalytic sites, producing an overall improvement of both FE and current density compared to the reference systems without POCs (see Tables S2 and S3). In most cases, these effects are predominantly interfacial rather than arising from reactions within the intrinsic cavity. Nevertheless, even when the role of the cavity is absent or uncertain, it can still enhance catalytic performance, as observed in hybrid systems with MNPs and in porphyrin‐based cages. While often attributed to local concentration effects, recent studies point to a more complex picture: host‐guest interactions, including the incorporation of cations, charged functionalities, or fullerene guests (e.g., C_70_) [147, 148, 149, 150], can further improve performance by stabilizing intermediates or inducing structural changes, such as increased rigidity upon guest binding. Overall, catalytic behavior arises from a combination of interfacial effects (e.g., CO_2_ accumulation and restricted diffusion at the POC‐catalyst interface, leading to enhanced interaction with active sites), host‐guest interactions, and structural factors, rather than from a single dominant mechanism. Disentangling the respective contributions remains a significant challenge for future studies (Figure 11).

Looking ahead, the effective integration of CO_2_ capture and conversion will critically depend on precise control over the packing of cage‐based materials, whether as discrete cages or extended networks. At the same time, future developments must align with synthetic sustainability (requiring scalable, cost‐effective preparation routes) and long‐term chemical and structural stability (required for practical deployment). In this context, coupling selective POCs with reduction catalysts in composite or blended systems, or incorporating active POCs into gas separation membranes, may represent an effective strategy to enhance performance and enable integrated CO_2_ capture and transformation without compromising economic viability or scalability.

## Author Contributions

**Valeria Amendola**: conceptualization, funding acquisition, writing – original draft, writing – review and editing, project administration. **Sonia La Cognata**: writing – original draft, writing – review and editing.

## Conflicts of Interest

The Authors declare no conflicts of interest.

## Supporting information



The main features of the POCs reported in Section 2, together with the catalytic performance in CO_2_ reduction of the active POCs and hybrid systems discussed in Section 4, are summarized in Tables S1–S3 provided in the Supplementary Information.
**Supporting File**: anie72917‐sup‐0001‐SuppMat.pdf.

## Data Availability

The data that support the findings of this study are available from the corresponding author upon reasonable request.
