# Supplementary material for: Porous Organic Cages for CO2 Capture and Confined Reduction
Source: Angew Chem Int Ed Engl. 2026 May 28;65(28):e5880155. doi: 10.1002/anie.5880155 (PMC13340508; doi:10.1002/anie.5880155)
Supplement: Supplementary file 1 — The main features of the POCs reported in Section 2, together with the catalytic performance in CO2 reduction of the active POCs and hybrid systems discussed in Section 4, are summarized in Tables S1–S3 provided in the Supplementary Information. Supporting File: anie72917‐sup‐0001‐SuppMat.pdf. [file ANIE-65-e5880155-s001.pdf]

Supporting Information

Porous Organic Cages for CO_2 Capture and Confined Reduction

Valeria Amendola* and Sonia La Cognata[a,b]

[a] Department of Chemistry
University of Pavia
viale T. Taramelli 12, I27100 Pavia, Italy
E-mail: valeria.amendola@unipv.it

[b] INSTM
via G. Giusti 9, I50121 Firenze, Italy

The references in the following Supplementary Tables have already been cited in the main text, and follow the same numbering.

Table S1. A summary of POCs investigated for CO_2 adsorption.

Cage	SA_{BET} N_2, 77K (m^2 g^{-1})	Adsorbed CO_2		CO_2/N_2 Selectivity	Temperature (K)	Pressure (bar)	CO_2 Q_{st} (kJ mol^{-1})	Ref.
		cm^3 g^{-1}	mmol g^{-1}					
**CC2**	30	8.7[a]		8.70[b]	293	1	-	54
**CC3**	436	39[a]		7.80[b]	293	1	-	54
**POC-3$_{cr}$**	1680		5.01	12.8[c]	273	1	25.5	56
				14.2[d]				
**POC-3$_{am}$**	1014		4.10	21.5[c]	273	1	24.2	56
				34.8[d]				
**POC-4**	1157	90	4.2	-	273	1	-	79
		50	2.01		298			
**POC-5**	1056		3.98	-	273	1	21.1-23.2	80
			~2.5		298	1		

[a] STP: standard temperature and pressure, i.e. 273.15 K and 1 bar; [b] ideal selectivity: ratio of the amounts adsorbed of the individual gases at a given pressure (q_A/q_B); [c] IAST (Ideal Adsorbed Solution Theory) selectivity, mixture N_2/CO_2 80/20 ; [d] Henry selectivity: adsorption in the zero-coverage limit ($K_{H,A}/K_{H,B}$).

Table S2. A summary of cage-based systems investigated for CO_2 catalytic reduction.

Type of catalysis	Sample	CO_2 reduction product	Conversion performance[a]	Ref.
Electrochemical conversion	Cu-nr/CC3	C_{2+} products	FE_{C2+} 76.1 %, j 1.7 A cm^{-2} (at -0.9 V)	137
	Cu-nr	C_{2+} products	FE_{C2+} 64.5 %, j 0.25 A cm^{-2} (at -0.9 V)	137
	Ag/CC3	CO	FE_{CO} 95.3 %, j_{CO} 0.39 A cm^{-2} (at -1.1 V)	137
	Ag-np	CO	FE_{CO} 85.8 %, j_{CO} 0.16 A cm^{-2} (at -1.1 V)	137
	Bi_2O_3/CC3	$HCOO^-$	FE_{HCOO} 77.1 %, j_{HCOO} 0.28 A cm^{-2} (at -1.1 V)	137
	Bi_2O_3-ns	$HCOO^-$	FE_{HCOO} 51.0 %, j_{HCOO} 0.14 A cm^{-2} (at -1.1 V)	137
Photochemical conversion	Au/DTE-POC-open	CO	Selectivity 78%, product amount 617 μmol, yield rate 56 μmol g^{-1} h^{-1}	140
	Au/DTE-POC-open/closed	CO	Selectivity 85.1%, product amount 1178 μmol, yield rate 105.18 μmol g^{-1} h^{-1}	140
	Pd-np/CC3	CH4	Selectivity ~98%, product amount 1.96 μmol, yield rate 78.5 μmol g^{-1} h^{-1}	141
	Pd-np	CH4	Selectivity ~97%, product amount 0.26 μmol, yield rate 10.2 μmol g^{-1} h^{-1}	141

[a] FE: faradaic efficiency; j: current density at the given potential (E vs RHE).

Table S3. A summary of porphyrin cage-based systems investigated for CO_2 catalytic reduction

Type of catalysis	Sample	CO_2-to-CO conversion performance[a]	Ref
Electrochemical conversion	FePB	FE_{CO} 100 %, j_{CO} 0.49 mA cm^{-2} (-0.63 V vs RHE), TON 6280, TOF 1.74 s^{-1}	142
	FeTPP	FE_{CO} 96%, j_{CO} 0.22 mA cm^{-2} (-0.63 V vs RHE), TON 3380, TOF 0.94 s^{-1}	142
	[(1)Fe]/C	FE_{CO} 90.8% (-1.55 V vs Ag/AgCl), overpotential ~1.4 V vs Ag/AgCl (at 5 mA cm^{-2}), 0.5 M $KHCO_3$	145
	FeTPP/C	FE_{CO} 50% (-1.55 V vs Ag/AgCl), overpotential ~1.3 V vs Ag/AgCl (at 5 mA cm^{-2}), 0.5 M $KHCO_3$	145
	FePB-2(P)	FE_{CO} 80%, j_{CO} 4 mA cm^{-2} (-2.17 V vs Fc/Fc^+), TON 2656	146
	CPOC-Co	FE_{CO} 92%, j_{CO} 18 mA cm^{-2} (-0.9 V vs RHE)	147
	TPP-Co	FE_{CO} 90%, j_{CO} 13.4 mA cm^{-2}, (-0.9 V vs RHE)	147
	$(FeCl)_2$BiCage	FE_{CO} 81%, j_{CO} 7.80 mA cm^{-2} (-0.64 V vs RHE), TOF_{CO} 58.09 h^{-1} (2500 nmol cm^{-2} Fe loading)	148
	C70@$(FeCl)_2$ BiCage*	FE_{CO} 96%, j_{CO} 10.86 mA cm^{-2} (-0.64 V vs RHE), TOF_{CO} 80.85 h^{-1} (2500 nmol cm^{-2} Fe loading)	148
	Co_2BiCage	FE_{CO} 72%, j_{CO} 9.5 mA cm^{-2} (-0.64 V vs RHE), TOF_{CO} 70.73 h^{-1} (2500 nmol cm^{-2} Co loading)	148
Photocatalytic conversion	FePB-2(P)	Selectivity 97%, TON 7006 (1168 per [Fe]), TOF 1429 min^{-1} (164 min^{-1} per [Fe])	146
	FeTPP	Selectivity 70%, TON 72 (12 per [Fe], TOF 1429 min^{-1} (0.08 min^{-1} per [Fe])	146

[a] FE: faradaic efficiency; j: current density at the given potential; TON: turnover number; TOF, turnover frequency.
